# A splicing variant of TFEB negatively regulates the TFEB-autophagy pathway

**DOI:** 10.1038/s41598-021-00613-y

**Published:** 2021-10-26

**Authors:** Jee-Yun Park, Hee-Young Sohn, Young Ho Koh, Chulman Jo

**Affiliations:** Division of Brain Disease Research, Department for Chronic Disease Convergence Research, Korea National Institute of Health, 187 Osongsaengmyeong2-ro, Osong-eup, Cheongju-si, 28159 Chungcheongbuk-do Korea

**Keywords:** Biochemistry, Cell biology, Molecular biology, Neuroscience

## Abstract

Transcription factor EB (TFEB) is a master regulator of the autophagy-lysosomal pathway (ALP). Here, we cloned a novel splicing variant of TFEB, comprising 281 amino acids (hereafter referred to as small TFEB), and lacking the helix-loop-helix (HLH) and leucine zipper (LZ) motifs present in the full-length TFEB (TFEB-L). The TFEB variant is widely expressed in several tissues, including the brain, although its expression level is considerably lower than that of TFEB-L. Intriguingly, in cells stably expressing small TFEB, the expression profile of genes was inverted compared to that in cells ectopically expressing TFEB-L. In addition, fisetin-induced luciferase activity of promoter containing either coordinated lysosomal expression and regulation (CLEAR) element or antioxidant response element (ARE) was significantly repressed by co-transfection with small TFEB. Moreover, fisetin-mediated clearance of phosphorylated tau or α-synuclein was attenuated in the presence of small TFEB. Taken together, the results suggest that small TFEB is a novel splicing variant of TFEB that might act as a negative regulator of TFEB-L, thus fine tuning the activity of ALP during cellular stress.

## Introduction

Autophagy is a cellular degradation process by which cytoplasmic constituents such as misfolded proteins, defective organelles, or invaded microorganisms are degraded via the lysosome^1,2^. Autophagy can be divided into several steps, beginning with the initiation of the phagophore and followed by elongation and closure of a double membrane structure around the cargo, forming an autophagosome which encapsulates cytosolic substrates^1^. The autophagosome then fuses with lysosomes to form an autophagolysosome that hydrolyzes its content^2^. The degraded content is subsequently released in the form of amino acids, lipids, and glycosides, which can be recycled. Autophagy is regulated by a series of proteins collectively referred to as autophagy-related gene (ATG) proteins, including ATG5, ATG8 (LC3), and ATG9^3^. Genetic deletion of ATG5 or ATG7, which is involved in autophagosome biogenesis, leads to neurodegeneration in mice^4,5^, indicating a fundamental role of this process in neuronal physiology and survival.

Transcription factor EB (TFEB) is a transcription factor of the microphthalmia family (MiT family), which includes MITF, TFE3, and TFEC^6,7^. These transcription factors share an identical basic region, which is required for DNA binding, and highly similar helix-loop-helix (HLH) and leucine zipper (LZ) motifs that are important for heterodimerization^8^. TFEB is a master regulator of lysosomal function and autophagy, orchestrating the expression of genes involved in lysosomal biosynthesis and function, autophagy, and lysosomal exocytosis^7,9^. Recent work has shown that TFEB also plays an important role in organelle biogenesis and metabolic processes^10,11^. Given its functional importance in cells, TFEB activity is strictly regulated through post-translational modifications, protein–protein interactions, and spatial organization^7^. In the normal resting state, TFEB is largely sequestered in the cytosol, but translocates to the nucleus under starvation conditions or lysosomal dysfunction^12,13^. Nuclear TFEB induces the expression of its target genes, which are involved in the autophagy-lysosomal pathway (ALP) and cellular metabolism, by binding to the coordinated lysosomal enhancement and regulation (CLEAR) elements in the respective gene promoters^12,14^. To date, it has been established that homo- or heterodimerization of TFEB increases its transcriptional activity; however, the mechanism of regulation of TFEB by its splicing variant is yet to be elucidated.

Neurodegenerative diseases are characterized by the aggregation of amyloid-β (Aβ) and hyperphosphorylated tau, and α-synuclein in Alzheimer’s disease (AD) and Parkinson’s disease (PD), respectively. Interestingly, defective autophagy and endolysosomal function have been extensively documented as early events in AD. Autophagic vacuoles (AVs) and enlarged endosomes accumulate in the brains of AD patients and mouse models, especially in dystrophic neurites and synaptic terminals^15–17^, likely due to impaired AV retrograde transport, maturation, and lysosomal clearance^18–20^. Compelling evidence has shown that α-synuclein aggregation also disrupts the retrograde transport of AVs, thus impairing autophagosome maturation and fusion with lysosomes^21,22^. Owing to its involvement in cellular clearance pathways, TFEB is an appealing therapeutic target for neurodegenerative diseases, including AD and PD. Accumulating evidence has shown that TFEB overexpression or its pharmacological activation in cellular and mouse models of AD and PD can reduce protein aggregation and improve neurological functions^23–28^, indicating that proper regulation of TFEB activity is critical for maintaining healthy neurons.

Here, we identified a novel small TFEB splicing variant, lacking the HLH-LZ motifs present in full-length TFEB (TFEB-L). We describe the molecular properties of small TFEB and its implications for neurodegenerative diseases.

## Results

### A splicing variant of TFEB is expressed in diverse tissues

**Figure 1:**
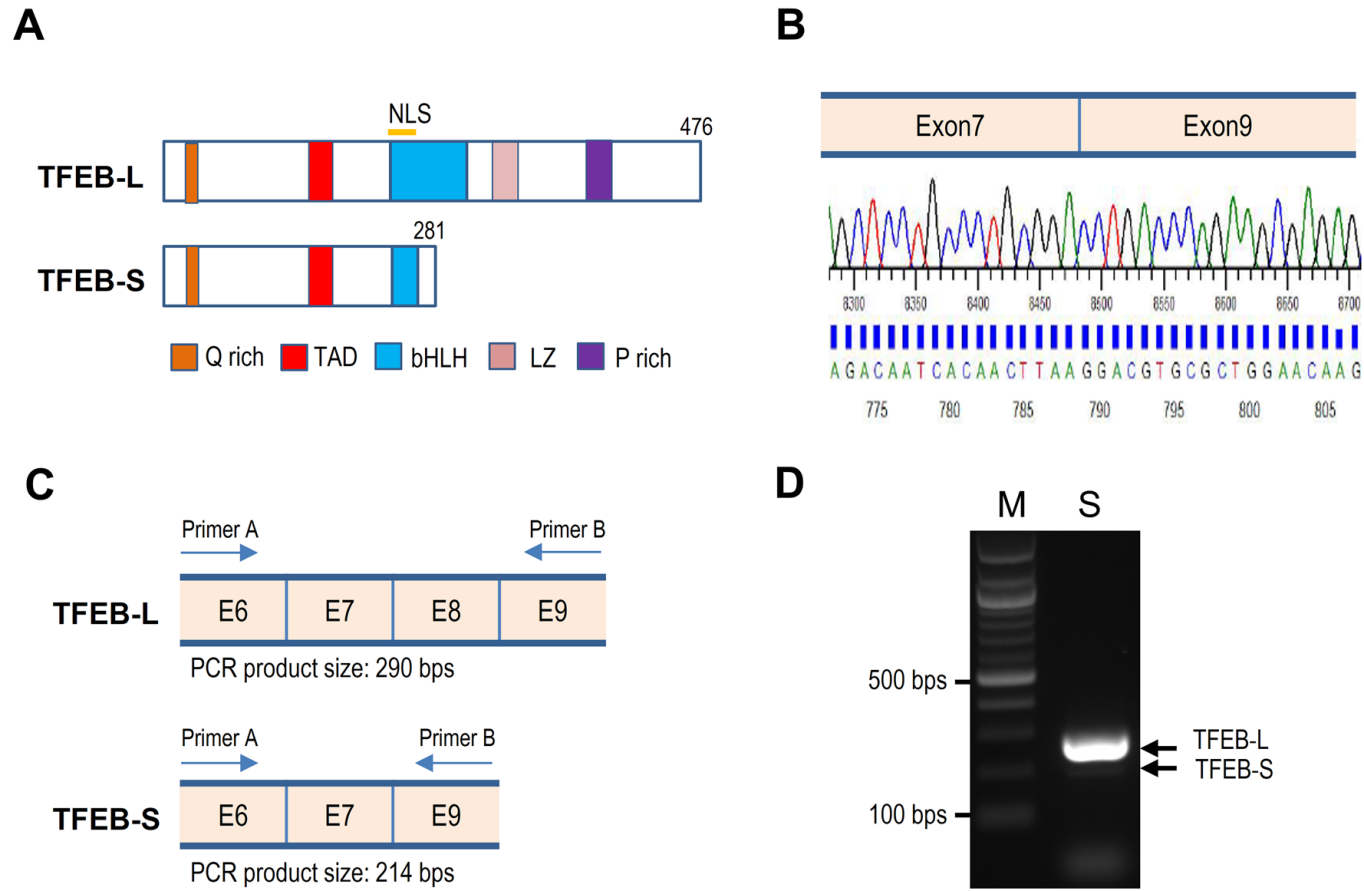
A small TFEB splicing variant lacking the helix-loop-helix and leucine zipper (HLH-LZ) region is present in human cell lines. (**A**) A schematic comparison of TFEB-L and small TFEB (TFEB-S). *Q rich* glutamine rich, *TAD* transactivation domain, *NLS* nuclear localization signal, *bHLH* basic helix-loop-helix, *LZ* leucine zipper, *P rich* proline rich. (**B**) The small *TFEB* cDNA cloned into the pHM6 plasmid was sequenced by an automatic sequencer. Exon 8 was skipped in the small *TFEB* splicing variant. (**C**) The position of primers (Supplementary Table 1) used for RT-PCR of *TFEB* cDNA and the expected sizes of the respective PCR products in the presence and absence of exon 8 are shown. (**D**) RT-PCR was performed using cDNA prepared from HEK293 cells, and the PCR product was analyzed on a 1.2% agarose gel.

During the cloning of the *TFEB* gene into the mammalian expression plasmid pHM6, we obtained a small *TFEB* splicing variant (TFEB-S) lacking HLH and LZ regions of TFEB-L (Fig. 1A). To determine how the small TFEB was derived from the *TFEB* gene, we sequenced the small *TFEB* variant cloned into pHM6. Compared to the *TFEB* gene, the small *TFEB* splicing variant was missing exon 8 (Fig. 1B). In addition, a nonsense mutation was introduced in exon 9 by a frameshift mutation due to an alternative splicing between exon 7 and exon 9 (Supplementary Fig. S1), generating a small TFEB protein of 281 amino acids (Supplementary Fig. S2). To confirm the isoform, we performed RT-PCR using a gene-specific primer set (Fig. 1C and Supplementary Table 1). As shown in Fig. 1D, a 214-bp PCR product was observed together with the main 290-bp fragment on a 1.2% agarose gel. To examine the expression of the small *TFEB* splicing variant in various tissues, we performed PCR using human cDNA panel prepared from various tissues. As shown in Supplementary Fig. S3, 214-bp PCR products were seen in most of human tissues. Together, the results suggest that the mRNA of small *TFEB* splicing variant is synthesized via an alternative splicing event in vivo.

**Figure 2:**
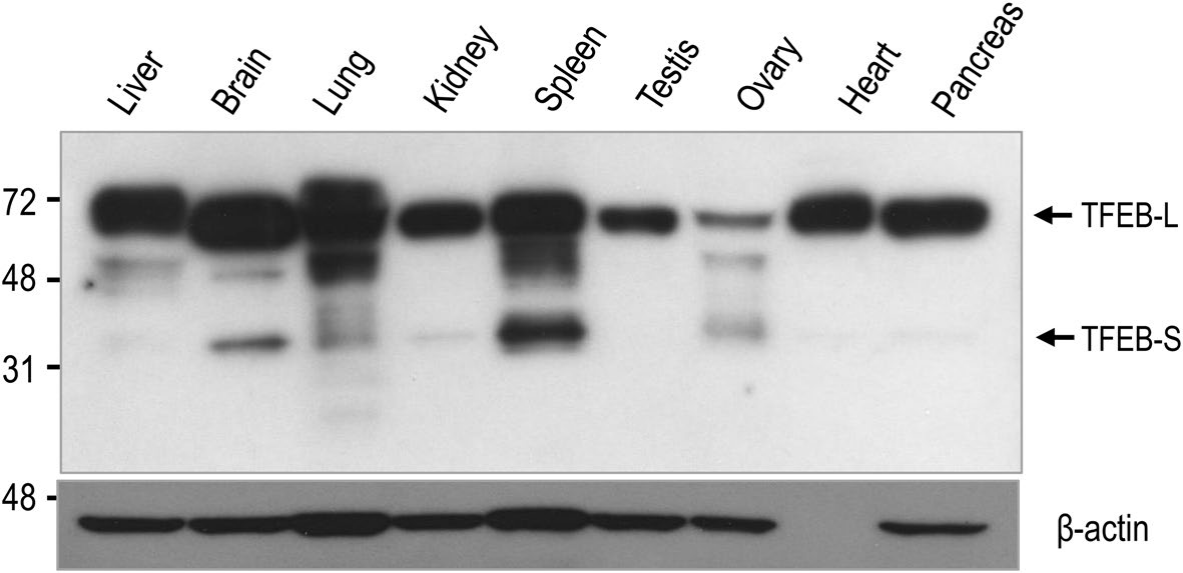
A small TFEB protein is produced in various human tissues. The expression level of TFEB and actin proteins was examined by immunoblotting using anti-TFEB and anti-actin antibodies, respectively. Full blots are provided in Supplementary Fig. S6.

To examine the protein expression of the small *TFEB* splicing variant in various tissues, we performed immunoblotting of diverse proteins from human tissues. Approximately 30-kDa protein bands corresponding to small TFEB were observed in several tissues, with the highest expression level in the spleen and a moderate expression level in the brain. In contrast, the expression level of small TFEB was barely detectable in the liver and testis (Fig. 2).

**Figure 3:**
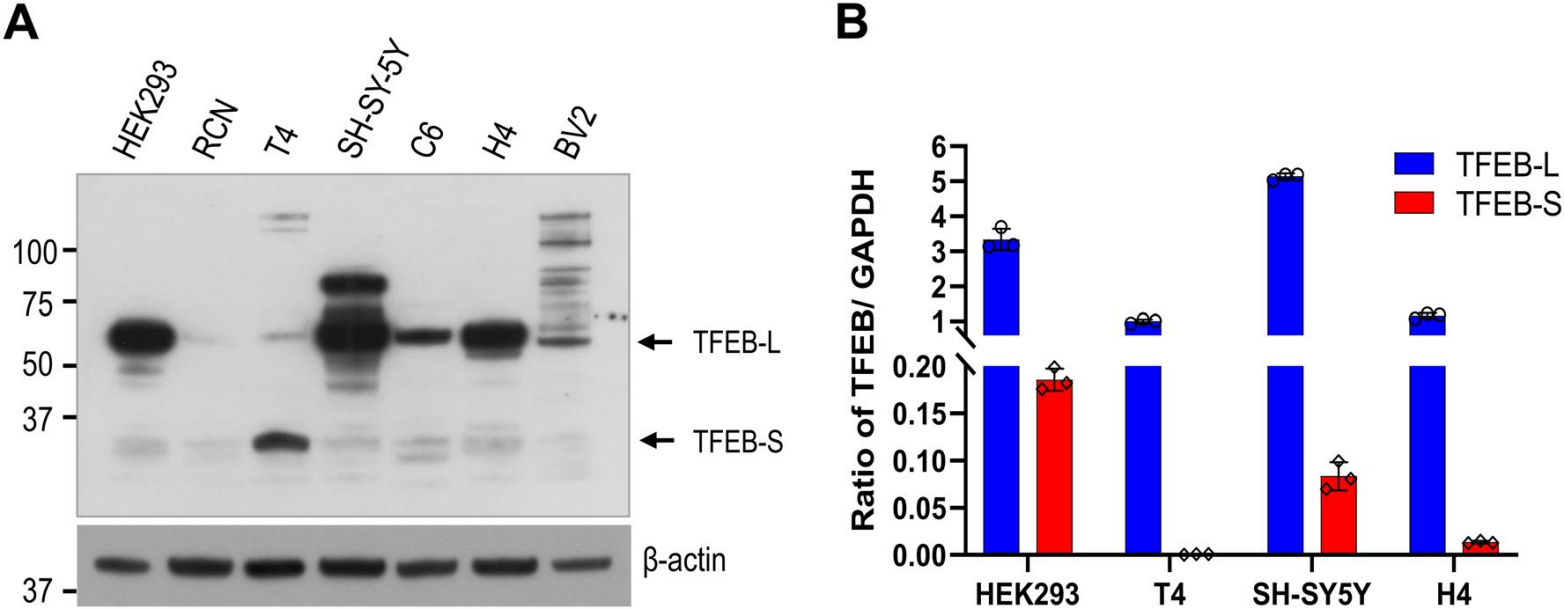
A small TFEB protein is produced in various cell lines. (**A**) The expression level of TFEB and actin proteins was examined by immunoblotting using anti-TFEB and anti-actin antibodies, respectively. Full blots are provided in Supplementary Fig. S6. (**B**) The expression levels of TFEB-L and small TFEB (TFEB-S) mRNA in several cell lines were analyzed by qRT-PCR using specific primer pairs for each gene.

To examine whether small TFEB is expressed in different cell lines, we analyzed its expression levels in cellular extracts using immunoblotting. As shown in Fig. 3A, most of the cells, including primary-cultured rat cortical neurons (RCN), express small TFEB. Interestingly, the expression level of small TFEB was highly increased in T4 neuronal cells. In addition, we compared the mRNA levels of small TFEB to those of TFEB-L using qRT-PCR. The mRNA levels of small TFEB and TFEB-L varied by cell line. Specifically, the mRNA level of small TFEB was 18-fold lower in HEK293 cells than that of TFEB-L. In contrast, the mRNA expression level was 1,396-fold lower in T4 neuronal cells (Fig. 3B), suggesting that the splicing event producing the small *TFEB* variant might be uncommon in normal states.

### Small TFEB is a negative regulator of the full-length TFEB

**Figure 4:**
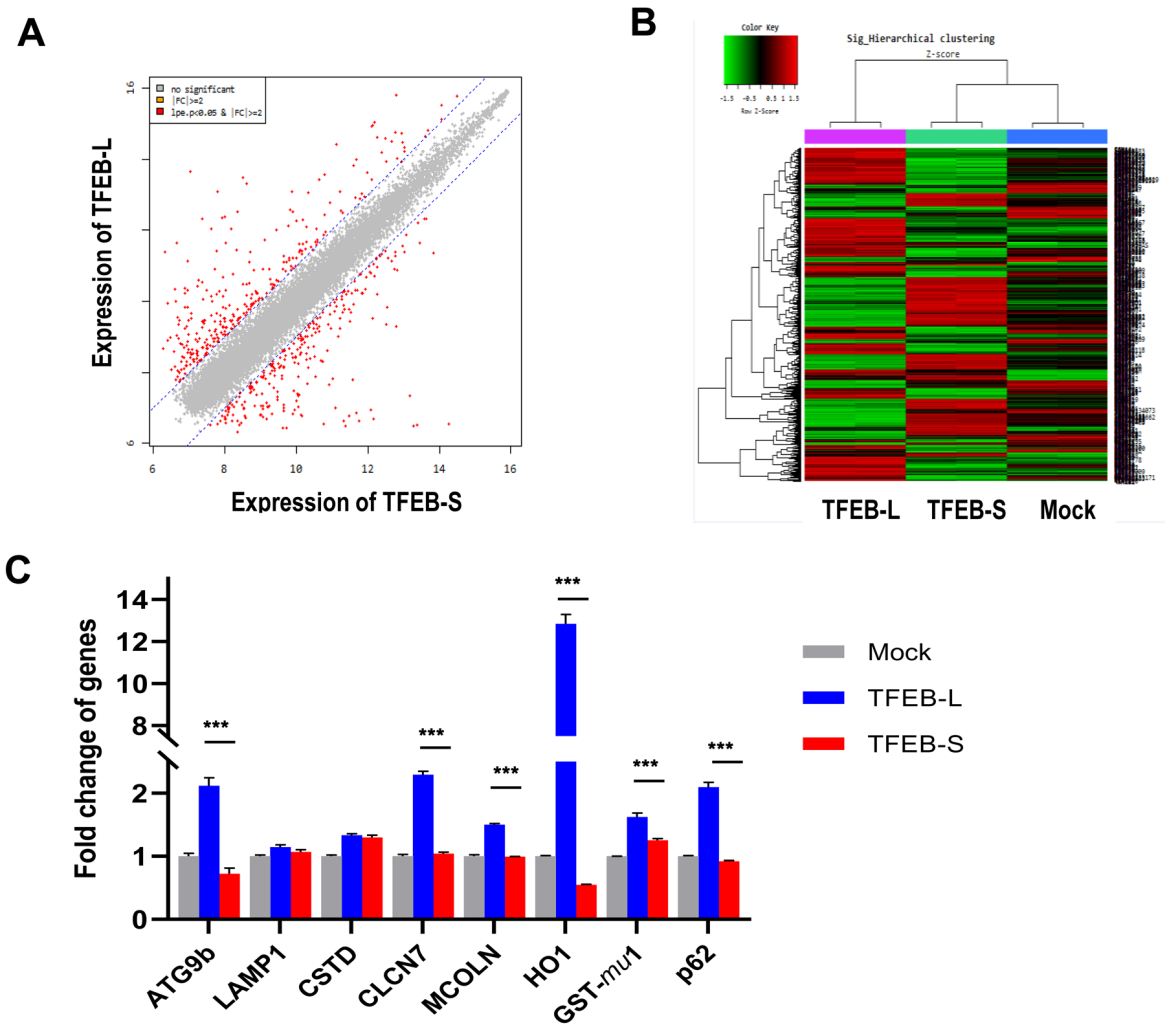
Small TFEB is a negative regulator of full-length TFEB. H4 cells stably expressing small TFEB (TFEB-S), or TFEB-L were analyzed using cDNA microarray. H4 cells stably transfected with the pcDNA3.1(+) plasmid served as mock controls. (**A**) A plot showing the expression levels of genes in stable TFEB-S and TFEB-L-expressing cells. (**B**) A heat map showing the expression levels of genes with a significant change. Shades of red represent upregulation and shades of green represent downregulation. (**C**) The mRNA levels of the autophagy-lysosomal pathway genes were analyzed by qRT-PCR. The bar graph represents the relative mRNA level of genes in small TFEB (TFEB-S) or TFEB-L-expressing cells compared to mock cells. The Data are shown as the mean ± S.E. of three independent experiments and were analyzed using Student’s *t* test. (***p < 0.001).

To examine the effect of small TFEB on the transcriptional activity of TFEB-L, we generated cell lines stably expressing small TFEB or TFEB-L. The gene expression profiles in small TFEB and TFEB-L-expressing cells were examined by cDNA microarray analysis as described in the Methods section. Interestingly, 541 genes showed more than a twofold increase or decrease in expression levels between the two cell lines, representing a relatively high proportion of genes (Fig. 4A). Notably, genes showing increased expression levels in TFEB-L-expressing cells inversely exhibited decreased expression levels in small TFEB-expressing cells (Fig. 4B), and vice versa, suggesting that small TFEB might play an antagonistic role against TFEB-L.

To confirm this hypothesis, we examined the expression levels of genes related to the autophagy-lysosomal pathway (ALP) using qRT-PCR. The gene expression of *ATG9b*, a gene involved in the initiation of autophagy, was repressed in cells stably expressing small TFEB, compared to that in stable control (Mock) or TFEB-L-expressing cells (Fig. 4C). Interestingly, the expression levels of *CLCN7* and *MCOLN* genes, which are involved in lysosomal function, were increased in the cells stably expressing TFEB-L, but were not induced in cells expressing small TFEB (Fig. 4C). We previously reported that Nrf2 is activated in cells stably expressing TFEB^29^. Thus, we also examined the expression of genes downstream of Nrf2, including *p62/SQSTM1* and *heme oxygenase* (*HO*)-1. As expected, the expression of genes downstream of Nrf2 was significantly induced in cell lines expressing TFEB-L; in contrast, the expression of *p62/SQSTM1* and *heme oxygenase* (*HO*)-1 was suppressed in cells expressing small TFEB (Fig. 4C). Interestingly, while the protein level of small TFEB was much higher in T4 cells than that in HEK293 cells (Fig. 3A), the expression levels of ALP genes were lower in T4 cells than those in HEK293 cells (Supplementary Fig. S4A). Altogether, these results suggest that small TFEB plays a contrasting role to TFEB-L.

**Figure 5:**
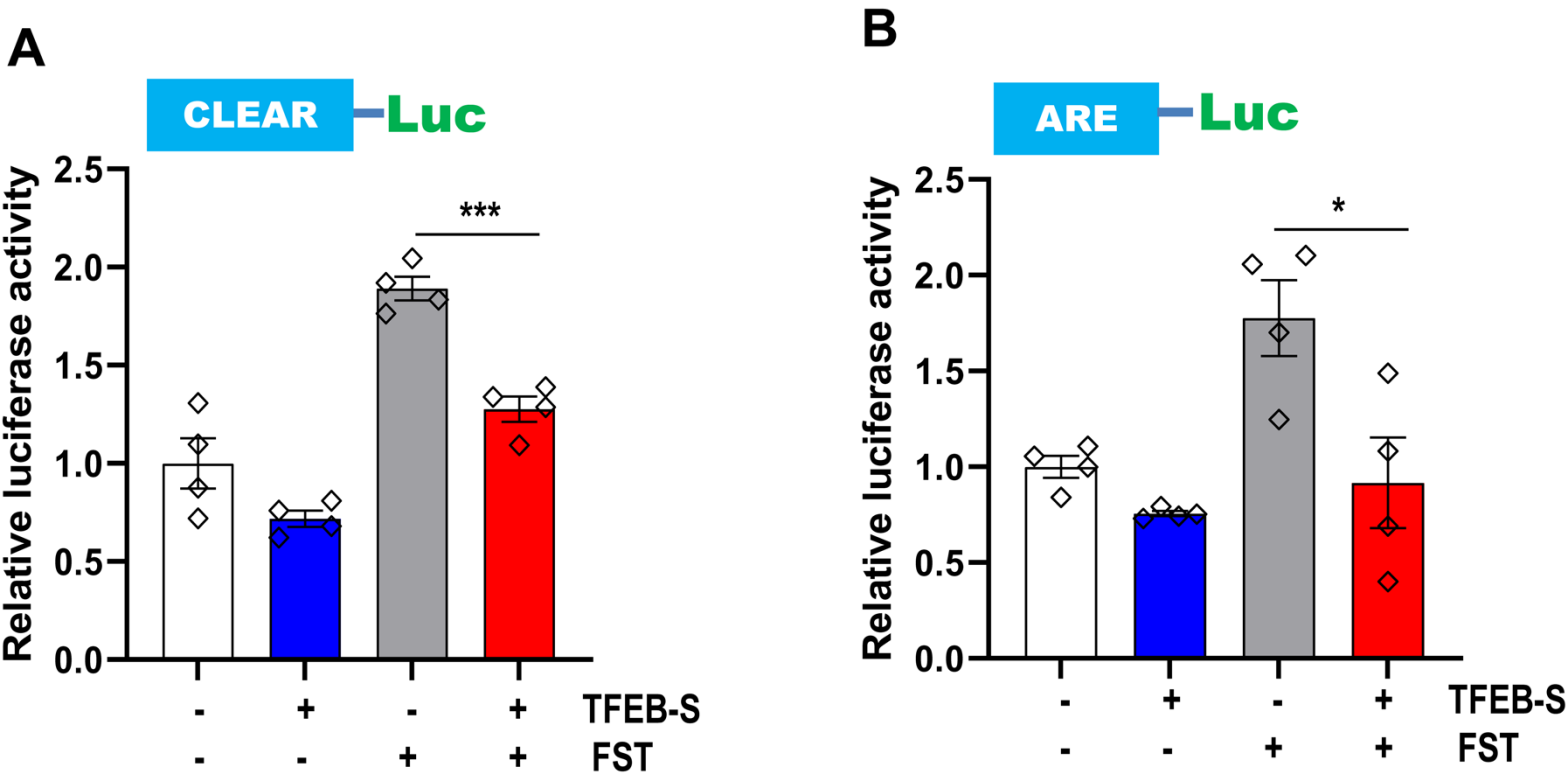
Small TFEB attenuates fisetin-induced transcriptional activity of TFEB and Nrf2. HEK293 cells were transiently co-transfected with either CLEAR-Luc or ARE-Luc reporter plasmids and either the plasmid expressing small TFEB variant or an empty vector. The cells were treated with 10 μM fisetin for 12 h, and assayed for the luciferase activity. The data are shown as the mean ± S.E. of three independent experiments and were analyzed using Student’s *t* test. (*p < 0.05; ***p < 0.001).

To further confirm whether small TFEB directly affects the transcriptional activity of TFEB-L, we performed a luciferase reporter assay following co-transfection of the luciferase reporter plasmid containing promoter with CLEAR or ARE elements and the small TFEB expression plasmid. We previously reported that fisetin, a small organic flavonoid, activates Nrf2 and TFEB in neuronal cells^30^. Thus, we examined luciferase activity in the cells expressing the promoters after 12 h of fisetin treatment. As expected, fisetin increased the transcriptional activity of the promoters containing either CLEAR or ARE elements; however, the increase in transcriptional activity was significantly ameliorated in the presence of small TFEB (Fig. 5). Also, we could observe a significant decrease of the promoter activity containing CLEAR in cells overexpressing small TFEB, compared with that in normal H4 cells (Supplementary Fig. S4B). Together, the results suggest that small TFEB likely negatively regulates the transcriptional activity of TFEB-L.

### Small TFEB co-localizes with the full-length TFEB in the nucleus

**Figure 6:**
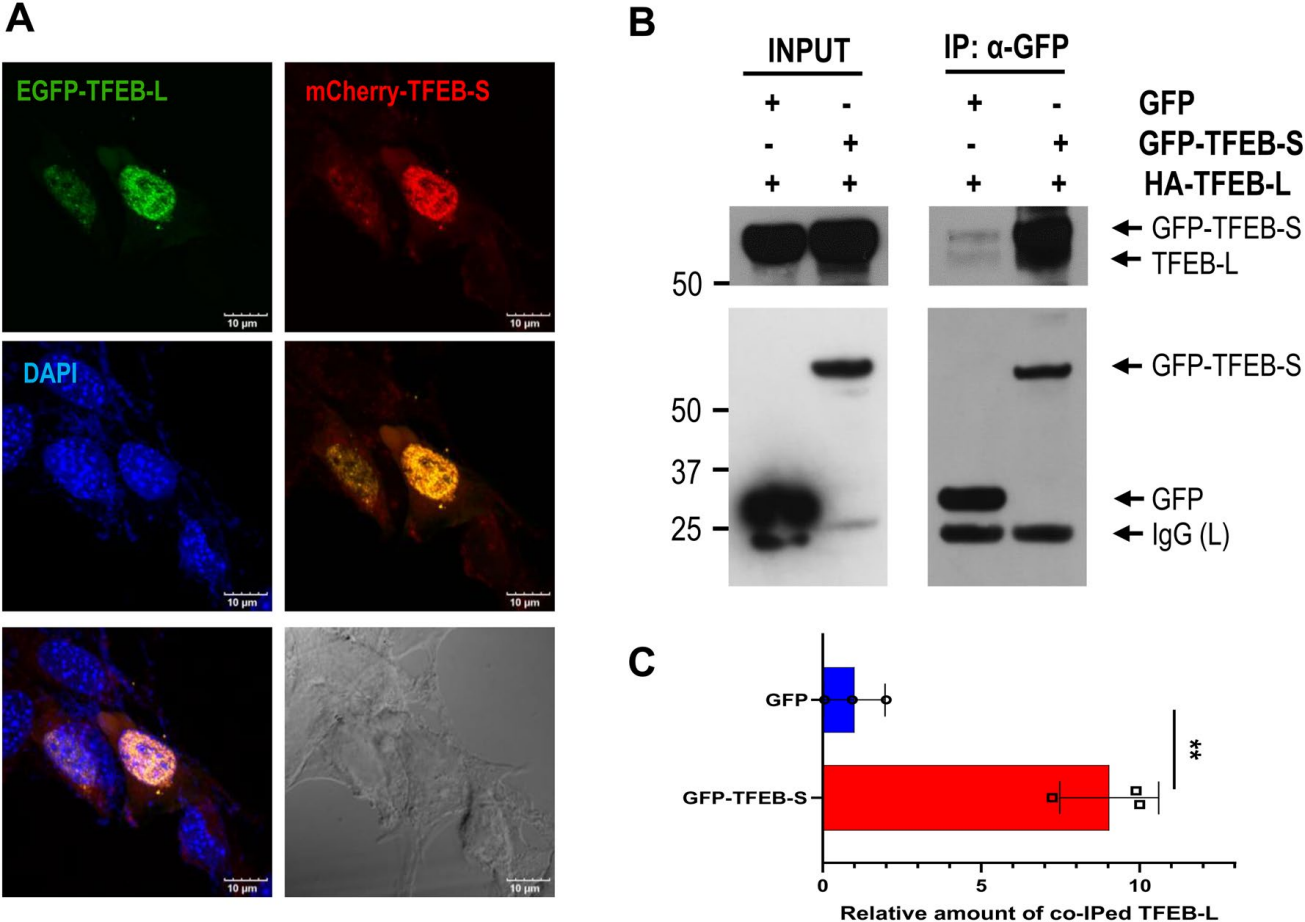
Activated small TFEB associates with full-length TFEB in the nucleus. (**A**) T4 cells were transiently co-transfected with EGFP-tagged TFEB (EGFP-TFEB-L) and mCherry-tagged small TFEB (mCherry-TFEB-S) and treated on the next day with 500 nM Torin1 for 1 h. Images were obtained using a confocal microscope (Olympus Corp., FV3000-ORS). (**B**) HEK293 cells were transiently co-transfected with the HA-TFEB expression plasmid and either the plasmid expressing EGFP-tagged small TFEB (GFP-TFEB-S) or the mock plasmid expressing only the GFP protein. The cells were treated with 500 nM Torin1 for 10 min. The cell lysates were used for small TFEB immunoprecipitation using an anti-GFP antibody. The protein level of small TFEB co-immunoprecipitated with TFEB-L was examined by immunoblotting using an anti-TFEB antibody. Full blots are provided in Supplementary Fig. S6. (**C**) Bar graph represents the relative amount of TFEB-L co-immunoprecipitated with small TFEB. The data are shown as the mean ± S.E. of three independent experiments and were analyzed using Student’s *t* test. (**p < 0.01).

To determine how small TFEB attenuates the transcriptional activity of TFEB-L, we first examined its cellular localization using a green fluorescence protein (GFP)-tagged TFEB-L and mCherry-tagged small TFEB protein, given that cellular localization is important for TFEB activity. When ectopically expressed in neuronal cells, TFEB-L was localized in the nuclei in about 50% cells, whereas small TFEB was found in the cytoplasm. However, when the cells were treated with either Torin1 or fisetin, both of which are mTORC1 inhibitors, small TFEB as well as TFEB-L translocated into the nucleus (Supplementary Fig. S5). Notably, the localization of small TFEB and TFEB-L overlapped in the nucleus, when observed by confocal microscopy as described in the Methods section (Fig. 6A), indicating that small TFEB and TFEB-L co-localized in the nucleus.

To further confirm the association between small TFEB and TFEB-L in the nucleus, we tested whether small TFEB co-precipitated with TFEB-L. Intriguingly, no interaction between small TFEB and TFEB-L was observed in the normal state. However, small TFEB co-immunoprecipitated with TFEB-L in cells treated with Torin1 for 10 min (Fig. 6B,C), suggesting that an interaction between small TFEB and TFEB-L in the nucleus might be involved in small TFEB-mediated repression of TFEB-L.

### Small TFEB attenuates the clearance of phosphorylated tau and α-synuclein

**Figure 7:**
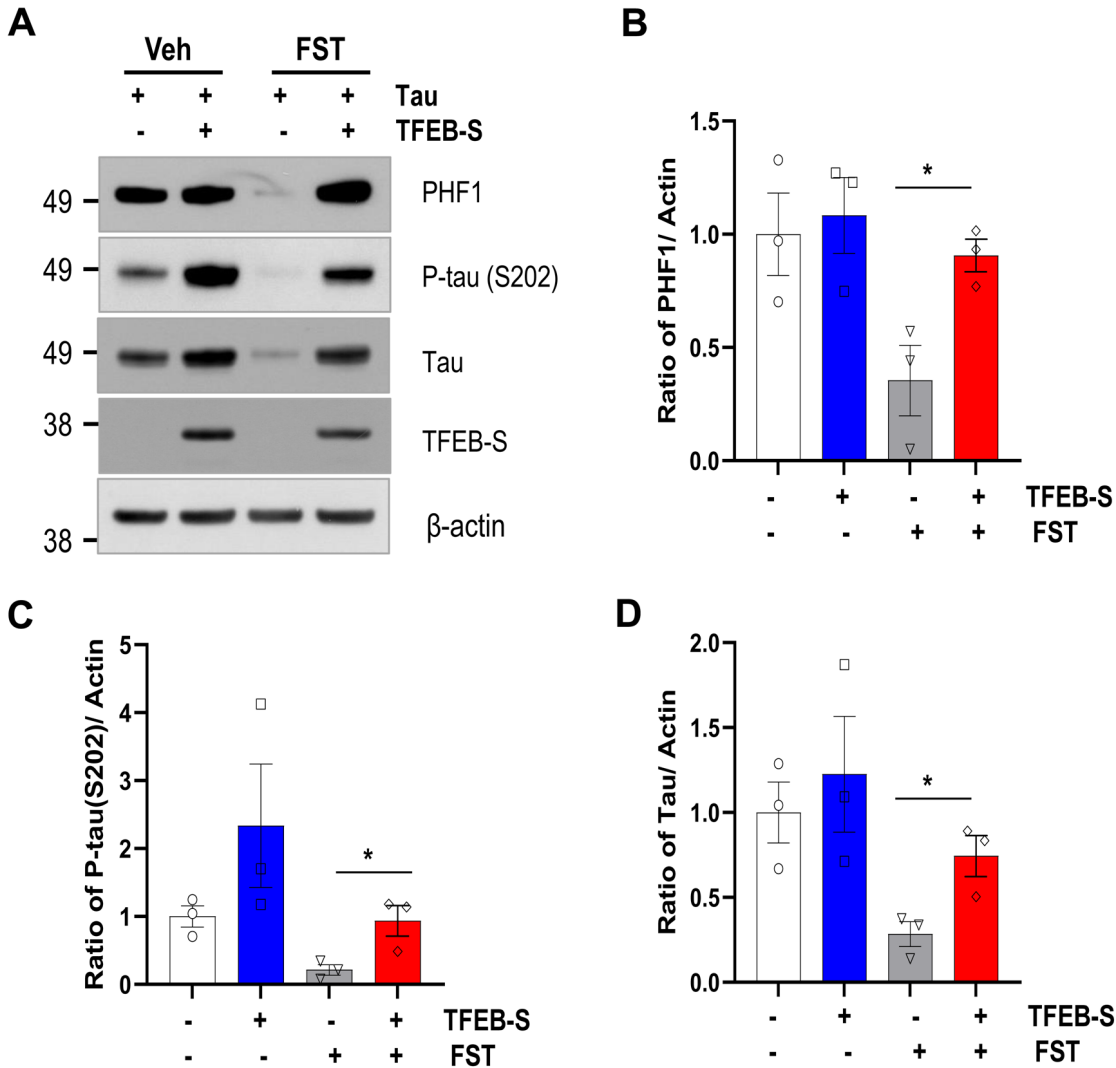
Small TFEB inhibits fisetin-induced clearance of phosphorylated tau. HEK293 cells were transiently co-transfected with the plasmid expressing tau and either the plasmid expressing small TFEB or an empty vector. The cells were treated with 10 μM fisetin for 12 h. (**A**) The protein levels of PHF1, phospho-tau (S202), total tau, small TFEB (TFEB-S), and actin were analyzed by immunoblotting using anti-PHF1, anti-phospho-tau (P-tau, S202), anti-tau, anti-HA and anti-actin antibodies, respectively. Full blots are provided in Supplementary Fig. S6. (**B**–**D**) Bar graphs represent the relative ratio of PHF1 (**B**), phospho-tau (S202) (**C**), and total tau (**D**) normalized with that of actin. The data are shown as the mean ± S.E. of three independent experiments and were analyzed using Student’s *t* test. (*p < 0.05).

**Figure 8:**
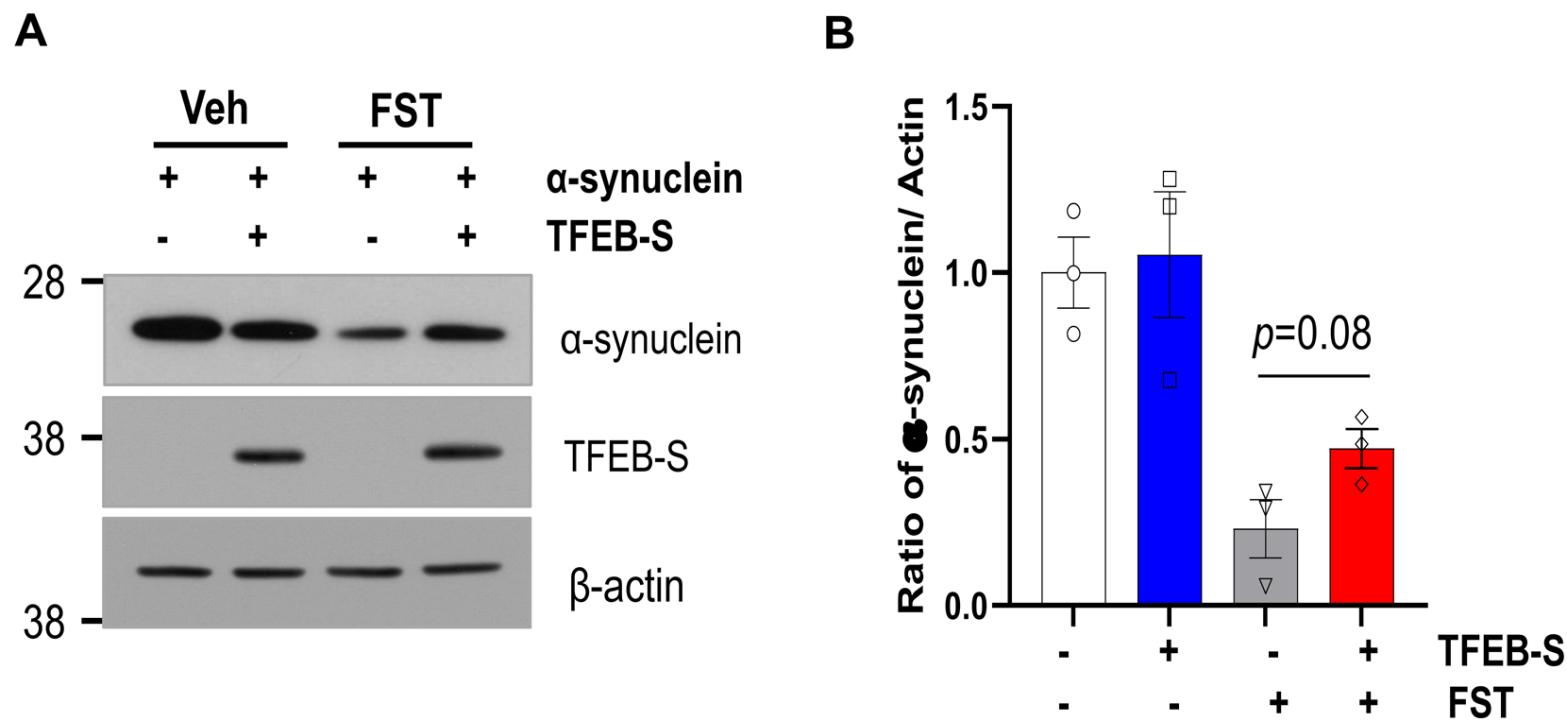
Small TFEB ameliorates fisetin-induced clearance of α-synuclein. HEK293 cells were transiently co-transfected with the plasmid expressing α-synuclein and either the plasmid expressing small TFEB or an empty vector. The cells were treated with 10 μM fisetin for 12 h. (**A**) The protein levels of α-synuclein, small TFEB (TFEB-S), and actin were analyzed by immunoblotting using anti-Myc, anti-HA and anti-actin antibodies, respectively. Full blots are provided in Supplementary Fig. S6. (**B**) Bar graph represents the relative ratio of α-synuclein normalized with that of actin. The data are shown as the mean ± S.E. of three independent experiments and were analyzed using Student’s *t* test.

TFEB enhances the clearance of pathological proteins such as phosphorylated tau and α-synuclein in neurons^24,27^. We previously observed that fisetin promotes the clearance of phosphorylated tau by activating TFEB and Nrf2 transcriptional factors^30^. Therefore, we examined the effect of small TFEB on fisetin-induced degradation of phosphorylated tau and α-synuclein. Intriguingly, the decrease in the levels of phosphorylated tau in the presence of fisetin was almost blocked by co-expression of small TFEB (Fig. 7), and the decrease in the levels of α-synuclein was attenuated in the presence of small TFEB (Fig. 8). The results suggest that small TFEB likely attenuates fisetin-induced TFEB activation, strongly supporting the notion that small TFEB acts as a negative regulator of TFEB-L.

## Discussion

The MiT family transcriptional factors contain several conserved functional domains, including the transactivation domain (AD), bHLH-LZ domain, and a proline-rich motif^8,31^. MiT family members can heterodimerize with one another^32^ and exhibit a large degree of overlap of their function and regulatory mechanisms^33,34^. Interestingly, isoforms lacking a particular domain or motif might play a role in the functional regulation of other MiT transcriptional factors. For example, multiple splicing variants of TFE3 were found to act as weak transactivators or repressors^35,36^. In case of TFEB, one study identified seven splicing variants of TFEB, with an alternative 5’ exon in the *TFEB* gene, which were apparently non-coding except for one variant^37^. Although TFEB expression levels differed according to tissues, all variants encoded the same TFEB protein^37^. Here, we report for the first time a novel small transcript lacking exons 8–10 which are present in the C-terminus of TFEB-L. As shown in Figs. 4, 5, 7, and 8, the small TFEB is likely a negative regulator of TFEB-L, which fine tunes the transcriptional activity of TFEB in vivo.

TFEB is comprised of an activation domain that synergistically activates transcription, a DNA-binding basic region, and a HLH-LZ region required for dimerization^7,34^ (Fig. 1A). Mutational analysis has revealed that the LZ region of TFEB is essential for high-affinity DNA binding, indicating that DNA binding is dependent on hetero- or homodimerization via the LZ domain^38^. Among the MiT family transcriptional factors, TFEC lacks the common activation domain required for transcriptional activation^39^. Therefore, it has been reported that TFEC is involved in the inhibition, rather than activation, of its downstream genes. TFE3 has a proline-rich motif in the C-terminus, which has also been confirmed to be another transactivation motif^35^. As shown in Fig. 1A, the small TFEB variant identified in this study does not contain the LZ domain and the proline-rich motif. Therefore, it is speculated that, like TFEC, small TFEB functions as a negative regulator. The molecular activity of small TFEB against TFEB-L, as shown in the present study, is in line with that deduced from its protein structure.

The gene expression profile of a cell line stably expressing small TFEB was reversed compared to that in cells expressing TFEB-L (Fig. 4). In addition, the expression of small TFEB attenuated the activity of promoters containing ARE and CLEAR elements following fisetin treatment (Fig. 5). Given that Nrf2 activity is dependent on that of TFEB^29^, the reduction in promoter activity containing ARE elements in the presence of the small TFEB variant likely originated from the repression of the transcriptional activity of TFEB. Previously, we reported that fisetin could enhance the degradation of phosphorylated tau through the activation of TFEB^30^. Therefore, we examined whether small TFEB affected the clearance of phosphorylated tau and α-synuclein. Notably, ectopic expression of small TFEB not only inhibited the clearance of phosphorylated tau, but also ameliorated the degradation of α-synuclein (Figs. 7 and 8). These results are also consistent with the previous results (Figs. 4 and 5), and thus strongly support the notion that the novel small TFEB is a negative regulator of TFEB-L.

mTORC1 kinase has been shown to phosphorylate specific serine residues (S122, S142, and S211) in TFEB, which helps to sequester TFEB in the cytoplasm^12,40–43^. In the present study, small TFEB protein was localized in the cytoplasm in normal states, and in the nuclei of cells treated with these mTORC1 inhibitors (Fig. 6A and Supplementary Fig. S5). A recent study suggested that a C-terminal serine-rich motif (S462/463/466/467/469) of TFEB is phosphorylated by mTORC1, which in turn enhances the nuclear localization of TFEB^44^, indicating that TFEB regulation by mTORC1 is plastic and dependent on cellular context^34^. Palmieri et al. suggested that AKT phosphorylation of TFEB at S467 also helps to trap TFEB in the cytoplasm, independent of mTORC1^45^. Given that small TFEB lacks the C-terminal serine-rich region, the nuclear localization of TFEB-L might be due to reduced activity of AKT or due to a complex interaction of these factors, including mTORC1.

The number of small TFEB transcripts was extremely low relative to that of TFEB-L (Fig. 3B). In T4 neuronal cells, the protein level of small TFEB was high, but its mRNA level was relatively low compared to that in other cells (Fig. 3A,B). This is likely due to a feedback regulation that maintains cellular homeostasis. In addition, when the plasmid expressing small TFEB was transfected into HEK293 cells, its protein level was in some cases too low to detect using immunoblotting, suggesting that small TFEB is highly unstable and rapidly degradable. TFEB protein is reported to be stabilized by phosphorylation at a C-terminal serine-rich motif (S462/463/467/469) by PKCβ^46^. Therefore, the destabilization of small TFEB could be elicited by the lack of the C-terminal region in which TFEB-L is stabilized through phosphorylation by PKCβ. Generally, most regulators should be unstable for proper, timely regulation in various cellular contexts. Thus, the instability of the small TFEB isoform may allow for finely tuned regulation of TFEB activity, and may be crucial for the proper spatio-temporal functioning of TFEB.

Overwhelming evidence supports a crucial role of ALP in the degradation of misfolded, aberrant proteins that accumulate in neurodegenerative diseases such as AD and PD^47–50^. Thus, enhancing ALP expression is a promising therapeutic strategy for the treatment of neurodegenerative diseases^51^. TFEB has emerged as a major regulator of ALP in neurons by coordinating autophagy induction and lysosomal biogenesis^52^. In contrast, TFEB dysregulation is thought to be involved in the pathology of neurodegenerative diseases^53^. A growing body of evidence indicates that TFEB can be regulated by phosphorylation with a variety of kinases, including mTORC1, GSK-3β, ERK2, PKCβ, and MAP4K3^34,54^. Small agonists of the TFEB pathway have been shown to ameliorate metabolic syndrome and extend the lifespan in mice and *C. elegans*, respectively^55^. Therefore, the activity of TFEB could be regulated by an agonist or a specific inhibitor or kinase activator, making TFEB an attractive therapeutic target for neurodegenerative diseases. Here, we identified, for the first time, a small molecular regulator of TFEB. Given its molecular function, it seems that the expression and molecular activity of small TFEB are critical for the function of TFEB in vivo, and in the etiology of neurodegenerative diseases. Moreover, our results suggest that the novel, small TFEB might be a promising therapeutic target for neurodegenerative diseases.

## Methods

### Antibodies, reagents, and plasmids

Anti**-**TFEB (4220), anti-phospho-tau (S202, 11834), anti-HA (2367), and anti-Myc (2276) antibodies were purchased from Cell Signaling Technology. Anti-TFEB (ab2636) antibody was obtained from Abcam. Anti-tau polyclonal (A0024) and anti-GFP (11814460001) antibodies were obtained from Dako and Roche, respectively. Anti-PHF (paired helical filament)1 (phospho-tau at S396/404) antibody was provided by Dr. P. Davies. Anti-β-actin (A5316) antibody was obtained from Sigma. Protease inhibitor cocktail (P8340), Torin1 (475991) and fisetin (5016) were purchased from Sigma, Calbiochem, and Tocris, respectively. Human cDNA panel prepared from various tissues was obtained from Clontech. The plasmid expressing human tau protein was previously used in the study^30^. The plasmid expressing Myc-tagged human α-synuclein was kindly gifted from Dr. S.J. Lee. Plasmids expressing human TFEB-L and small TFEB were constructed by cloning the human *TFEB* gene, which was amplified from cDNA prepared from human neuroglioma (H4) cells, into the *Kpn*1 and *EcoR*1 sites of the pHM6 plasmid (Roche). To generate the plasmid expressing EGFP-tagged TFEB-L, the *TFEB* gene was subcloned into the *EcoR1* and *BamH1* sites of the pEGFP-C1 plasmid. The small *TFEB* gene was subcloned into the *EcoR1* and *Xho1* sites of the pmCherry plasmid (Clontech) to generate the plasmid expressing an mCherry-tagged small TFEB. To generate the luciferase reporter CLEAR-Luc plasmid, an oligomer containing 3 × CLEAR element sequence was cloned into the *Kpn1* and *BglII* sites of the pGL4.14 vector (Promega). All plasmids were confirmed by sequencing. The ARE-Luc plasmid was constructed as described in a previous study^29^.

### Cell culture and generation of stable cell lines

HEK293, SH-SY5Y, rat glial (C6), and mouse microglial (BV2) cells were purchased from ATCC. Human neuroglioma (H4) and immortalized mouse cortical neuronal (T4) cells were used in the previous studies^56,57^. Human neuroglioma (H4), HEK293, SH-SY5Y, rat glial (C6), and mouse microglial (BV2) cells were cultured in Dulbecco's modified Eagle's medium (DMEM) supplemented with 10% fetal bovine serum (FBS), 10 U/mL penicillin, and 100 U/mL streptomycin, at 37 °C in a humidified atmosphere containing 5% CO_2_. Immortalized mouse cortical neuronal cells (T4) were cultured in DMEM supplemented with 10% FBS, 0.1% hygromycin, 10 U/mL penicillin, and 100 U/mL streptomycin, at 33 °C in a humidified atmosphere containing 5% CO_2_. Primary cell culture was conducted as previously described^30,57^. H4 cells stably expressing human TFEB-L and small TFEB were transfected with the pHM6-TFEB and pHM6-small TFEB plasmids, respectively, and established by G418 selection. Mock cells were established with pcDNA3.1(+) using the same method.

### Reverse transcription PCR (RT-PCR)

Total RNA was isolated form HEK293 cells using the RNAeasy Mini Kit (Qiagen) according to the manufacturer’s protocol. cDNA was synthesized from 2 μg of total RNA using RT-PCR EcoDry Premix (Clontech). PCR was performed on a PCR machine (Applied Biosystems). Each reaction consisted of 50 ng cDNA product, and 10 µM of primers (Supplementary Table 1) in the PCR premix (Bioneer, Korea). The reaction was incubated at 96 °C for 5 min, followed by 30 cycles of 96 °C for 15 s, 58 °C for 15 s and 72 °C for 30 s. After the reaction was completed, the product was electrophoresed on 1.2% agarose gel. The DNA image on the gel was taken on AlphaImager HP (Alpha Innotech).

### cDNA microarray

Microarray analysis was performed as described in a previous study^29^. Total RNA was extracted from H4 cells using TRIzol reagent (Invitrogen) according to the manufacturer’s protocol. Biotinylated cRNA (anti-sense RNA) was prepared using the TargetAmp-Nano Labeling Kit (Epicenter) according to the manufacturer’s instructions. Briefly, 500 ng of total RNA was reverse-transcribed into cDNA using a T7 oligo(dT) primer. Then, second-strand cDNA was synthesized and used to synthesize cRNA by in vitro transcription in the presence of biotin-NTP. After purification, cRNA was quantified using an ND-1000 spectrophotometer (NanoDrop). A total of 750 ng of labeled cRNA was hybridized to each Human HT-12 v4.0 Expression Beadchip (Illumina Inc.) for 18 h at 58 °C. The array signal was detected using Amersham fluorolink streptavidin-Cy3 (GE Healthcare Bio-Sciences) according to the bead array manual, and scanned with an Illumina Bead Array Reader Microarray Scanner, according to the manufacturer's instructions.

### Transient transfection and luciferase assays

Cells were transiently transfected with the relevant plasmids using Lipofectamine 2000 (Invitrogen). The total amount of DNA per well was normalized to the relevant mock vectors. For the luciferase assay, HEK293 cells were transiently co-transfected with pHM6-small TFEB and TK-renilla plasmids and either CLEAR-Luc or ARE-Luc reporter plasmids, and the cells were then treated with 10 μM fisetin for 12 h. Luciferase activity was measured using the Luciferase Assay System (Promega, E2920) and a luminometer (GLOMAX, Promega). The transfection efficiency was normalized to Renilla activity.

### Immunoblotting

Immunoblotting was performed as described in the previous study^29^. Briefly, cells were washed once with PBS and lysed with modified RIPA buffer (10 mM Tris–HCl [pH 7.4], 150 mM NaCl, 1 mM EGTA, 1% NP-40, 0.25% sodium deoxycholate, 0.1% SDS) containing phosphatase (1 mM NaF and 1 mM Na_3_VO_4_) and protease inhibitors. Proteins were extracted on ice with vortexing three times for 30 min and lysates were centrifugated at 10,000×*g* for 10 min at 4 °C. The supernatants were used for immunoblotting after boiling in 1 × SDS-sample loading buffer with reducing agent at 97 °C for 7 min. Protein concentration was analyzed by the BCA method (Sigma). Each protein sample (20–40 μg) was electrophoresed on 4–12% NuPAGE Bis–Tris Protein gels (Invitrogen) at a constant current of 20 mA, followed by transfer to NC (nitrocellulose) membranes (GE Healthcare). A membrane of protein blots from various human normal tissues (TB37-Set-1) was purchased from G-Biosciences. Each membrane was blocked with 5% skim milk, followed by immunoblotting with the indicated antibodies. The blots were developed using chemiluminescence (Thermo Scientific), and the images were analyzed using the ImageJ software.

### Quantitative real-time PCR (qRT-PCR)

qRT-PCR was performed as described in the previous study^29^. Briefly, total RNA was isolated from cells using the RNAeasy Mini Kit (Qiagen) according to the manufacturer’s protocol. Each cDNA was synthesized using the RT-PCR EcoDry Premix (Clontech) using 2 μg of total RNA. The qRT-PCR reaction was performed using a real-time PCR detection system (QuantStudio 6 Flex, Applied Biosystems). Each reaction consisted of 50 ng cDNA, 0.2 µM of primers (Supplementary Table 2), and 12.5 µL of SYBR Green Real-Time PCR Master Mix (Invitrogen, 4344463). PCR reaction was initiated in a 96-well plate at 95 °C for 10 min, followed by 40 cycles of 95 °C for 15 s and 60 °C for 1 min. After the reaction was completed, the threshold cycle (CT) was automatically recorded. CT was defined as the fractional cycle number at which the fluorescence signal passed the fixed threshold. All reactions were performed in triplicate for each sample.

### Fluorescent protein imaging

T4 cells were transiently co-transfected with EGFP- and mCherry-tagged TFEBs. On the next day, the cells were fixed with 4% paraformaldehyde solution for 20 min. The fixed cells were washed three times with PBS. The cover-slips were mounted on glass slides with ProLong gold antifade reagent (Invitrogen, P36935). Images were photographed using a confocal microscope (Olympus Corp., FV3000-ORS).

### Immunoprecipitation

Cells were lysed in NP-40 lysis buffer (0.5% NP-40, 10 mM Tris–HCl [pH 8.0], 150 mM NaCl, 10 mM sodium pyrophosphate, 1 mM EDTA) containing 1 mM NaF, 1 mM Na_3_VO_4_, and 1 × protease inhibitor cocktail. Equal amounts of lysates were incubated with 2 μg of antibodies pre-conjugated with sheep anti-mouse magnetic beads (DYNAL, Invitrogen) for 3 h on a rotational shaker at 4 °C. After an overnight incubation at 4 °C, the beads were washed three times with NETN wash buffer (0.1% NP-40, 50 mM Tris–HCl [pH 8.0], 150 mM NaCl, 1 mM EDTA) and boiled in 1 × SDS-sample loading buffer and 1 × reducing agent at 97 °C for 7 min. Proteins were separated by SDS-PAGE and immunoblotted as described above.

## Supplementary Information

Supplementary Information.
