# Supplementary material for: A splicing variant of TFEB negatively regulates the TFEB-autophagy pathway
Source: Sci Rep. 2021 Oct 26;11:21119. doi: 10.1038/s41598-021-00613-y (PMC8548335; doi:10.1038/s41598-021-00613-y)
Supplement: Supplementary file 1 — Supplementary Information. [file 41598_2021_613_MOESM1_ESM.pdf]

Supporting Information

A splicing variant of TFEB negatively regulates the TFEB-autophagy pathway

Jee-Yun Park[1], Hee-Young Sohn[1], Young Ho Koh[1], and Chulman Jo[1*]

[1]Division of Brain Disease Research, Department for Chronic Disease Convergence Research, Korea National Institute of Health, 187 Osongsaengmyeong2-ro, Osong-eup, Cheongju-si, Chungcheongbuk-do 28159, Korea

*Correspondence and requests for materials should be addressed to C.J.
E-mail: chulmanjo@gmail.com
Tell: +82-043-719-8631

Supplementary Figure S1

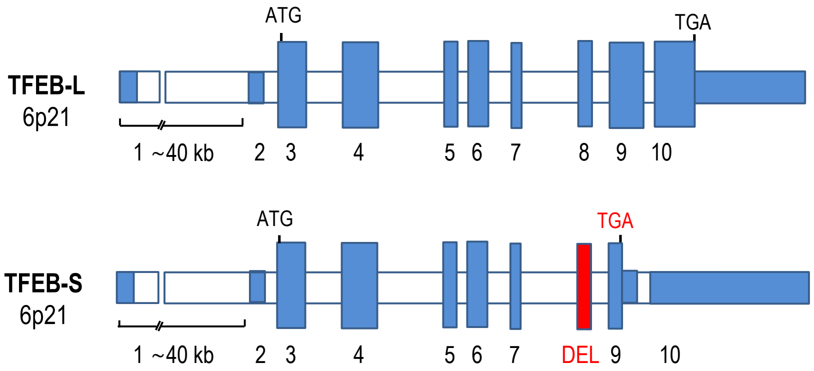

Fig. S1. Genetic organization of human *TFEB* gene.

Supplementary Figure S2

```
V1  MASRIGLRMQLMREQAQQEEQRERMQQQAVMHYMQQQQQQQQQQLGGPPTPAINTPVHFQ    60
V2  MASRIGLRMQLMREQAQQEEQRERMQQQAVMHYMQQQQQQQQQQLGGPPTPAINTPVHFQ    60
    ************************************************************

V1  SPPPVPGEVLKVQSYLENPTSYHLQQSQHQKVREYLSETYGNKFAAHISPAQGSPKPPPA   120
V2  SPPPVPGEVLKVQSYLENPTSYHLQQSQHQKVREYLSETYGNKFAAHISPAQGSPKPPPA   120
    ************************************************************

V1  ASPGVRAGHVLSSSAGNSAPNSPMAMLHIGSNPERELDDVIDNIMRLDDVLGYINPEMQM   180
V2  ASPGVRAGHVLSSSAGNSAPNSPMAMLHIGSNPERELDDVIDNIMRLDDVLGYINPEMQM   180
    ************************************************************

V1  PNTLPLSSSHLNVYSSDPQVTASLVGVTSSSCPADLTQKRELTDAESRALAKERQKKDNH   240
V2  PNTLPLSSSHLNVYSSDPQVTASLVGVTSSSCPADLTQKRELTDAESRALAKERQKKDNH   240
    ************************************************************

V1  NLIERRRRFNINDRIKELGMLIPKANDLDVRWNKGTILKASVDYIRRMQKDLQKSRELEN   300
V2  NLRTCAGTRAPSSRPLWITSGGCKRTCKSPGSWRTTLAAWR-------------------   281
    **

V1  HSRRLEMTNKQLWLRIQELEMQARVHGLPTTSPSGMNMAELAQQVVKQELPSEEGPGEAL   360
V2  ------------------------------------------------------------   281

V1  MLGAEVPDPEPLPALPPQAPLPLPTQPPSPFHHLDFSHSLSFGGREDEGPPGYPEPLAPG   420
V2  ------------------------------------------------------------   281

V1  HGSPFPSLSKKDLDLMLLDDSLLPLASDPLLSTMSPEASKASSRRSSFSMEEGDVL       476
V2  --------------------------------------------------------       281
```

V1, TFEB-L; V2, TFEB-S

Fig. S2. Amino acid sequence of human full-length and small TFEB.

Supplementary Figure S3

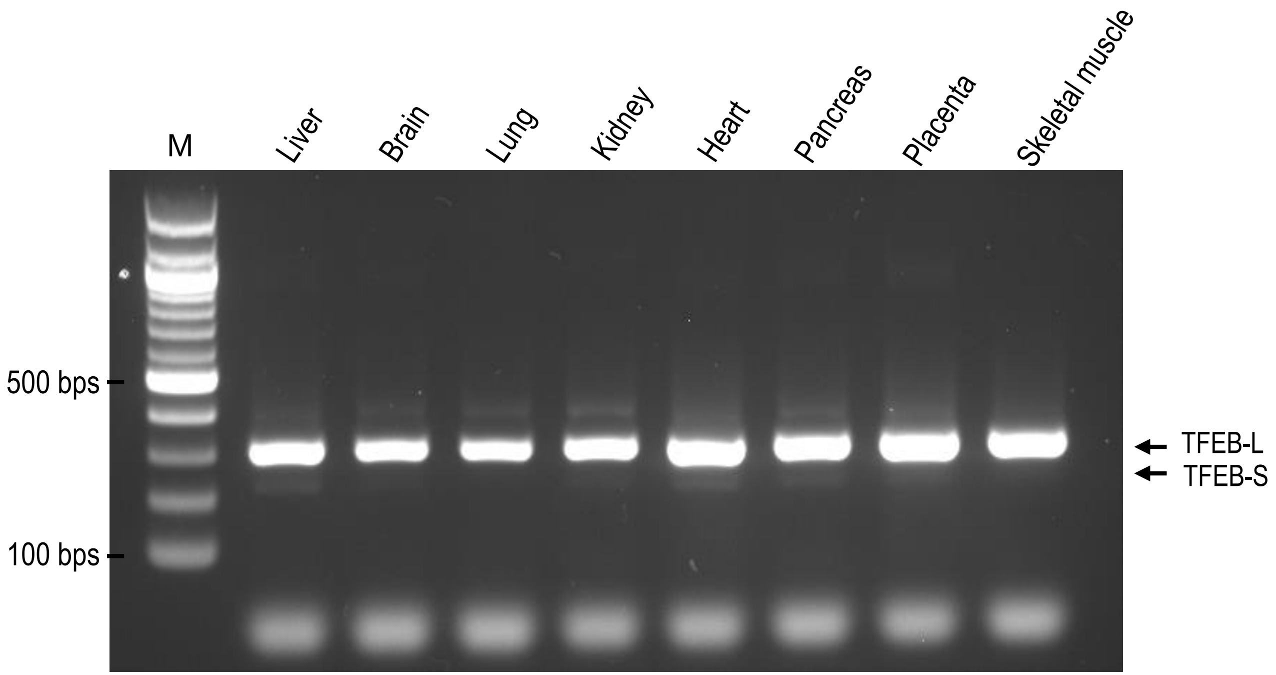

Fig. S3. A small, splicing variant of TFEB is produced in various human tissues. The expression of TFEB were examined by PCR using the primer pair (Supplementary Table 1) and cDNA prepared from various human tissues (Clontech). PCR was performed according to the protocol of RT-PCR as described in Methods. The PCR product was analyzed on a 1.2% agarose gel. M, *iVDye* 100 bp DNA ladder (GenDEPOT, Korea).

Supplementary Figure S4

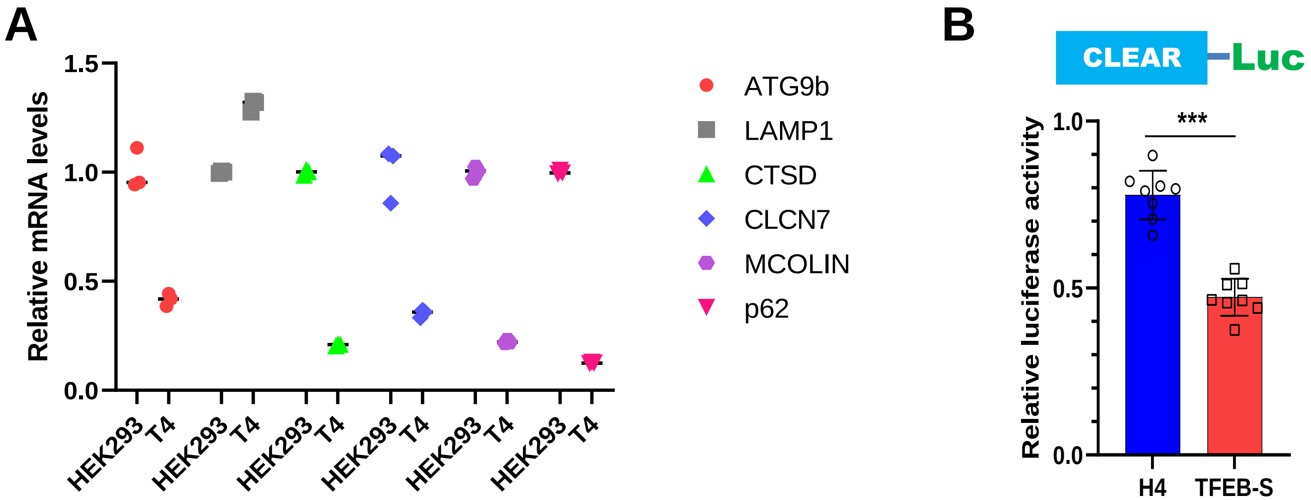

Fig. S4. Small TFEB is a negative regulator of full-length TFEB. (A) The expression levels of the autophagy-lysosomal pathway genes in HEK293 and T4 neuronal cells were analyzed by qRT-PCR using specific primer pairs for each gene (Supplementary Table 2). (B) H4 and H4 cells stably expressing small TFEB (TFEB-S) were transiently transfected with the CLEAR-Luc reporter plasmid. On the next day, cells were assayed for the luciferase activity. The data are shown as the mean ± S.E. of three independent experiments and were analyzed using Student's *t* test. (****p* < 0.001)

Supplementary Figure S5

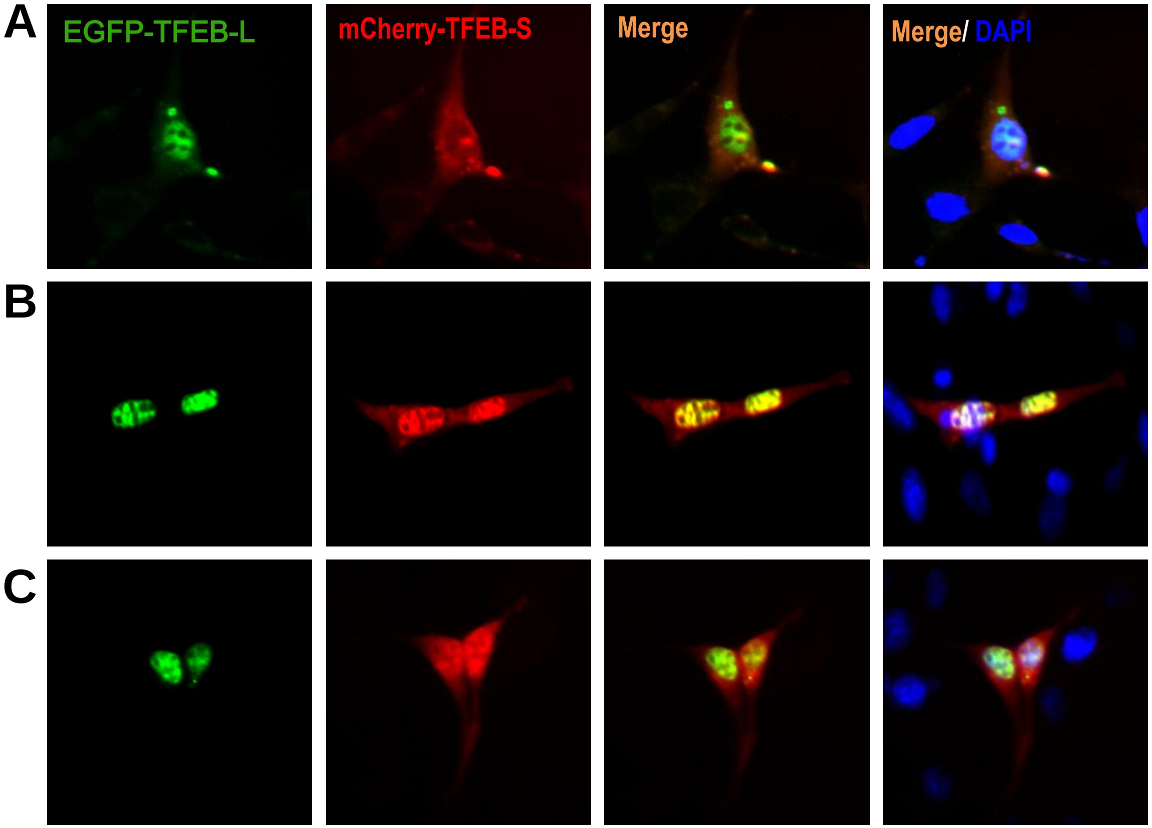

Fig. S5. Nuclear small TFEB co-localizes with full-length TFEB in nucleus. T4 neuronal cells were co-transfected with EGFP-tagged full-length TFEB and mCherry-tagged small TFEB. On the next day, the cells were treated with DMSO (A), 500 nM Torin (B), and 10 μM fisetin (C) for 1 h. The localization of TFEB in the cells was observed under fluororescence microscope.

Supplementary Figure S6: Full scans of uncropped gel and blots.

Fig. 1

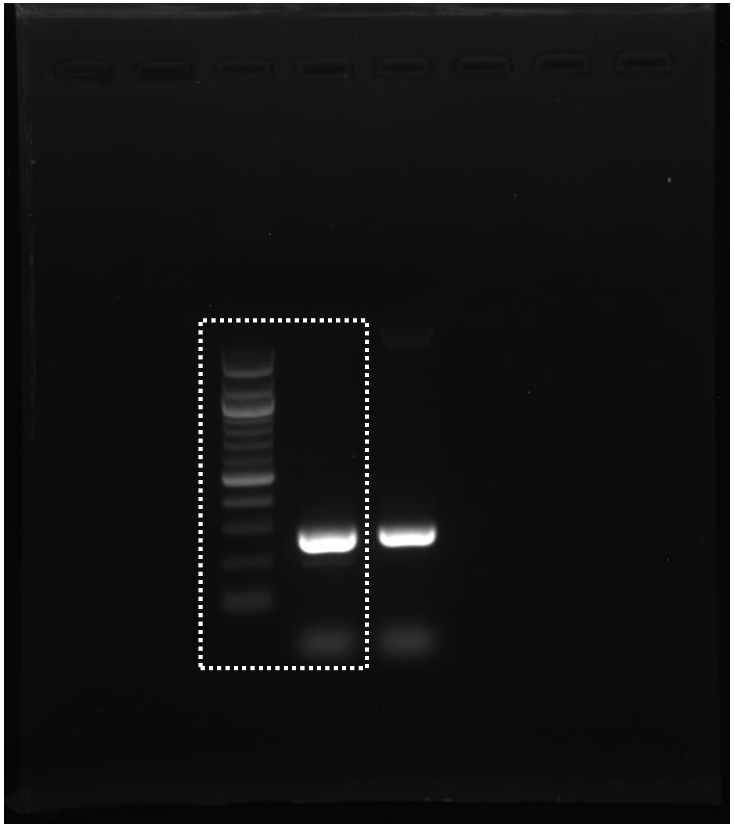

Supplementary Figure S6 continued.

Fig. 2

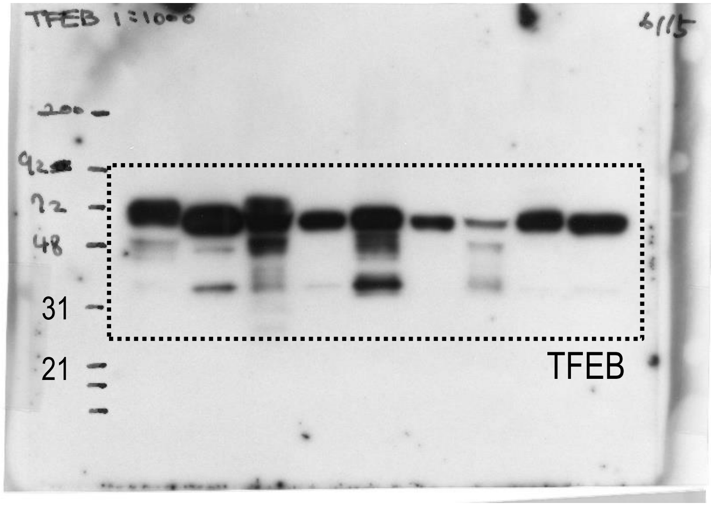

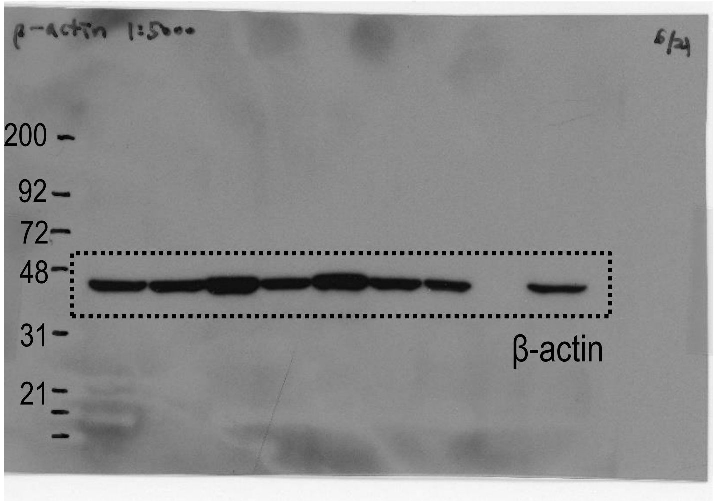

Supplementary Figure S6 continued.

Fig. 3A

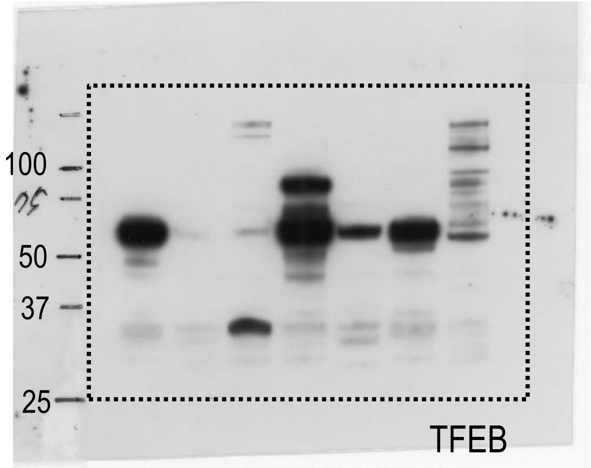

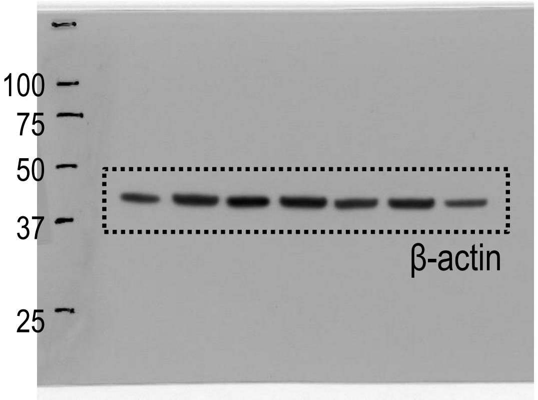

Supplementary Figure S6 continued.

Fig. 6B

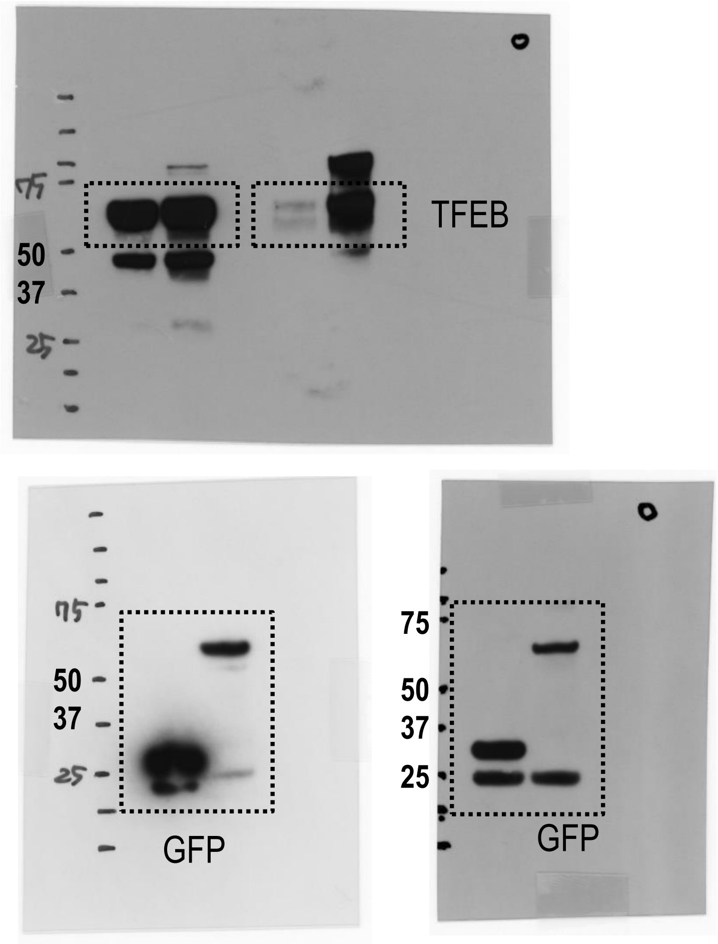

Supplementary Figure S6 continued.

Fig. 7A

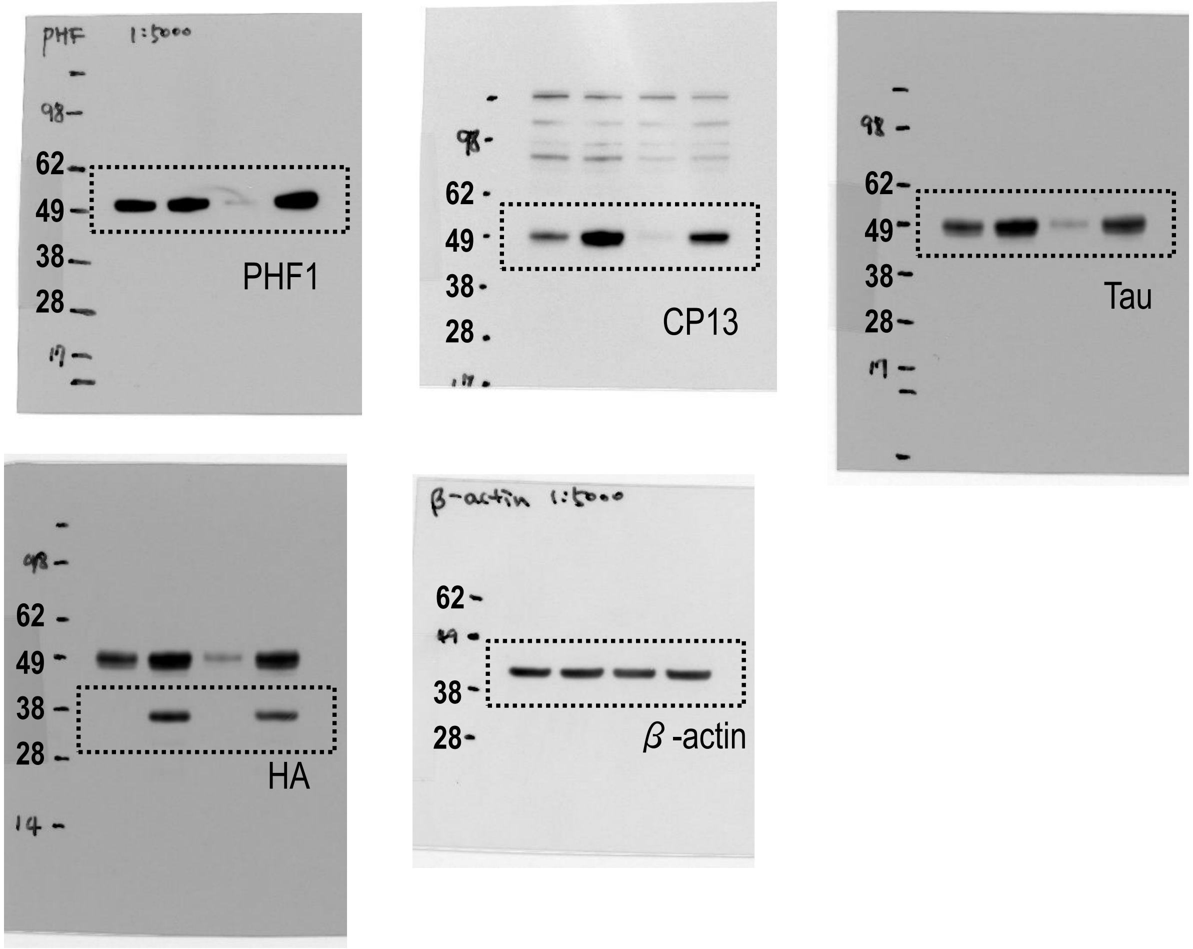

Supplementary Figure S6 continued.

Fig. 8A

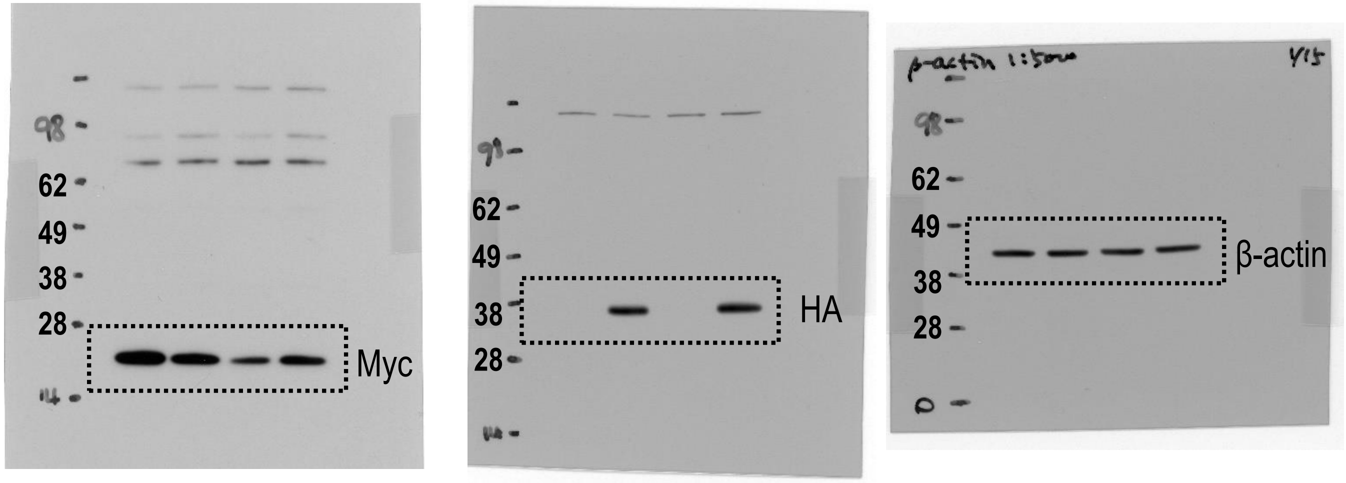

Supplementary Table 1 The sequence of RT-PCR primer pair used for confirming the small, alternative splicing variant of TFEB (Fig.1C).

Name	Sequence
Primer A	5'-CTGAATGTGTACAGCAGCGA-3'
Primer B	5'-CTCCGGATGTAATCCACAGA-3'

Supplementary Table 2 The sequence of primers used for qRT-PCR.

Gene	Forward	Backward
Human TFEB	5'-CTAACAGATGCTGAGAGCAGAG-3'	5'-CCAGCGCACGTCCTTAG-3'
Human small TFEB	5'-GAAAGACAATCACAACTTAAGGAC-3'	5'-TCCGGATGTAATCCACAGAG-3'
Human ATG9b	5'-TCCTTCGATGCGTGGATTAC-3'	5'-GCATCTGACAGGGTCACTTT-3'
Human LAMP1	5'-CGTCAGCAGCAATGTTTATGG-3'	5'-CATGTTCTTAGGGCCACTCTT-3'
Human cathepsin D	5'-AGTGCTTCACAGTCGTCTTC-3'	5'-GGACTTGTCGCTGTTGTACT-3'
Human CLCN7	5'-CAGGGTCATCAAGGGCAATA-3'	5'-GCTCTATGAAAGCCACAATCAC-3'
Human MCOLN	5'-CAAGAACCTCACGCTCAAATTC-3'	5'-GCAGTCCGGGATCTCATTATT-3'
Human GST-*mu*1	5'-CTTTCCCAATCTGCCCTACTT-3'	5'-CCACACGAATCTTCTCCTCTTC-3'
Human p62/SQSTM1	5'-ATTGAGTCCCTCTCCCAGAT-3'	5'- CGCTCCGATGTCAT AGTTCTT-3'
Human GAPDH	5'-GGTGTGAACCATGAGAAGTATGA-3'	5'-GAGTCCTTCCACGATACCAAAG-3'
Mouse TFEB	5'-CAGAAGCGAGAGCTAACAGATG-3'	5'-GAACCTGCGTCTTCTCTCAATTA -3'
Mouse small TFEB	5'-CTAACAGATGCTGAGAGCAGAG-3'	5'-CCAGCGCACGTCCTTAG-3'
Mouse GAPDH	5'-TCAACAGCAACTCCCACTCTTCCA-3'	5'- ACCCTGTTGCTGTAGCCGTATTCA -3'
